# MicroRNA-33 suppresses CCL2 expression in chondrocytes

**DOI:** 10.1042/BSR20160068

**Published:** 2016-05-06

**Authors:** Meng Wei, Qingyun Xie, Jun Zhu, Tao Wang, Fan Zhang, Yue Cheng, Dongyang Guo, Ying Wang, Liweng Mo, Shuai Wang

**Affiliations:** * Department of Nephrology and Rheumatology, Chengdu Military General Hospital. No. 270, Rongdu Avenue, Jinniu District, Chengdu, Sichuan, 610083, P.R. China; † Department of Orthopaedics, Chengdu Military General Hospital. No. 270, Rongdu Avenue, Jinniu District, Chengdu, Sichuan, 610083, P.R. China

**Keywords:** CCL2, chemotaxis, microRNA-33 (*miR-33*), microRNA-124 (*miR-124*), osteoarthritis, reporter gene

## Abstract

CCL2-mediated macrophage infiltration in articular tissues plays a pivotal role in the development of the osteoarthritis (OA). miRNAs regulate the onset and progression of diseases via controlling the expression of a series of genes. How the *CCL2* gene was regulated by miRNAs was still not fully elucidated. In the present study, we demonstrated that the binding sites of *miR-33* in the 3′UTR of *CCL2* gene were conserved in human, mouse and rat species. By performing gain- or loss-of-function studies, we verified that *miR-33* suppressed CCL2 expression in the mRNA and protein levels. We also found that *miR-33* suppressed the CCL2 levels in the supernatant of cultured primary mouse chondrocytes. With reporter gene assay, we demonstrated that *miR-33* targeted at AAUGCA in the 3′UTR of *CCL2* gene. In transwell migration assays, we demonstrated that the conditional medium (CM) from *miR-33* deficient chondrocytes potentiated the monocyte chemotaxis in a CCL2 dependent manner. Finally, we demonstrated that the level of *miR-33* was decreased, whereas the CCL2 level was increased in the articular cartilage from the OA patients compared with the control group. In summary, we identified *miR-33* as a novel suppressor of CCL2 in chondrocytes. The *miR-33*/CCL2 axis in chondrocytes regulates monocyte chemotaxis, providing a potential mechanism of macrophage infiltration in OA.

## Abstract (precis)

*miR-33* suppresses CCL2 production via directly targeting its 3′UTR in the chondrocytes. The *miR-33*/CCL2 axis in the chondrocytes regulates monocyte chemotaxis, providing a potential mechanism of macrophage infiltration in osteoarthritis (OA).

## INTRODUCTION

Osteoarthritis (OA) is a common rheumatic disease with irreversible destruction of the joint cartilage [1]. Pro-inflammatory cytokines, including IL-6 [2], TNF-α [3] and IL-1β [4], are potent inducers of OA development. Especially, the IL-1 receptor has been selected as a functional target for multiple inflammatory diseases like rheumatic arthritis, in mouse models and human subjects [5,6].

Macrophages are suggested to be the most important source of those pro-inflammatory cytokines [7–9]. Indeed, macrophage infiltration in the articular tissues is a fundamental pathology of OA progression [7,8]. Therefore, blocking the recruitment of macrophages represents an effective strategy for the prevention and therapy of OA [10,11].

CCL2, also termed as monocyte chemoattractant protein-1 (MCP-1), is a potent attractor of monocyte [12]. Blocking of MCP-1 displays notable improvement of multiple inflammatory diseases (e.g. OA) [13–15]. Therefore, it is rather important to investigate the regulatory mechanism of CCL2 expression. Generally, the production of pro-inflammatory cytokines and chemokines (e.g. CCL2) is under the tight control of several signalling pathways, like NF-κB, JNK or ERK [16]. Recently, miRNAs are found to be novel regulators of gene expression, displaying a great potential in disease therapy [17,18].

miRNAs are a class of non-coding RNAs (18–23 nucleotides) that negatively regulate mRNA stability and translation. In the past decade, miRNAs have been identified to regulate inflammatory signals and play pivotal roles in the regulation of bone biology [18,19]. Most recently, CCL2 is reported to be regulated by *miR-124* [20,21]. However, whether any other miRNAs involve in CCL2 expression is still not clear.

In the present study, we predicted the potential miRNAs which might target at the 3′UTR of CCL2, and further identified *miR-33* as a suppressor of CCL2 expression. We also investigated the role of *miR-33*/CCL2 axis in regulating monocyte chemotaxis, providing a potential mechanism of macrophage infiltration in chronic inflammation, such as OA.

## MATERIALS AND METHODS

### Isolation and culture of primary mouse chondrocytes

All the mouse experiments were conducted in accordance with the guidelines for the care and use of laboratory animals and were approved by the Animal Care and Use Committee in Chendu Military General Hospital. The male C57BL/6 mice were housed in a pathogen-free facility with a 12 h light, 12 h dark cycle. Primary mouse chondrocytes were isolated and cultured following the protocol as described in our recent study [17,22].

### Collection of human samples

All the experiments involving human subjects were approved by the ethics committee in Chendu Military General Hospital and the informed consent was obtained from all the subjects. Human articular cartilage samples were collected from the knee joints of patients undergoing the total knee replacement surgery due to OA (*n*=10, male, average age: 52.6 years) or trauma (*n*=6, male, average age: 45.2 years). Two to three pieces of sample tissues (2–3 mm in diameters) were dissected and immediately stored in liquid nitrogen for subsequent real-time PCR assay.

### Protein extraction and immunoblotting assay

Proteins were extracted with RIPA lysis buffer and quantified by the BCA kit (Roche). The proteins were separated by SDS/10% PAGE and transferred to a PVDF membrane for immunoblotting assay with the antibodies [Anti-GAPDH (#2118, Cell Signaling Technology) and Anti-CCL2 (ab8101, Abcam)].

### Real-time PCR

Total RNAs were isolated by Trizol reagent (Invitrogen) according to the manufacturer's protocol. RNAs were transcribed into cDNAs using Omniscript (Qiagen). Quantitative real-time PCR was performed using the 7900HT Fast Real-Time PCR system (Applied Biosystems). The mRNA expression levels were normalized to GAPDH. Reactions were done in duplicate using Applied Biosystems TaqMan Gene Expression Assays and Universal PCR Master Mix. The relative expression was calculated by the 2(^−ΔΔ^*^C^*_t_) method. All the primers used for PCR are available upon request.

*miRNA-33* expression level was detected with the TaqMan microRNA assay real-time fluorescent quantitative PCR technology (TaqMan®MicroRNA Assays, Life Technologies). The fluorescence quantitative PCR reaction system consisted of the following: 7.67 μl RNase-free H_2_O, 10 μl TaqMan Universal PCR Master Mix, 1.33 μl RT product and 1 μl TaqMan small RNA assay. The reaction condition was as follows: 95°C for 10 min, 35 cycles at 95°C for 15 s and 60°C for 60 s. Samples were normalized by U6 snRNA expression.

### Gain- or loss-of-function studies

Overexpression of *miR-33* was carried out by transfecting the chondrocytes with a *miR-33* mimic (MC12410, Thermo Fisher Scientific). Inhibition of *miR-33* was conducted by transfecting the chondrocytes with an anti-miRNA of *miR-33* (AM12410, Thermo Fisher Scientific). A scramble miRNA (AM17010, Thermo Fisher Scientific) was employed as control. For transfection of the chondrocytes, the working concentration of the miRNAs was 100 nM.

### Molecular cloning experiments

Those experiments for plasmid construction and site-directed reporter gene mutation were performed as described recently and previously [17,23].

### Transfection and reporter gene assays

The primary chondrocytes were plated in 12-well or 96-well culture plates (Corning Costar) at a density of 70–80% confluence. Then, the luciferase reporters (wt or mut) and miRNAs (*miR-33* mimic, anti-*miR-33* or scramble miRNA) were transfected using Lipofectamine 2000 according to the manufacturer's instructions (Invitrogen). Briefly, cells were incubated 3 h before transfection with foetal calf serum-free, antibiotic-free media and then transfected with the luciferase reporters (0.4 μg/ml) or miRNAs (100 nM). After transfection for 6 h, the media were removed and replaced with complete growth medium. After a further 24 h, cells were washed twice with PBS and lysed with specific reporter lysis buffer. Then, the luciferase activities of the cell lysate were evaluated according to the manufacturer's instructions (Promega), and the total protein concentration in each well was measured as an internal control. Transfection experiments were performed three times in triplicate. Data were represented as fold induction over reporter gene treated with scramble miRNAs.

### ELISA

CCL2 levels in the supernatant of cultured chondrocytes were measured with CCL2 (#MJE00) ELISA Kits from R&D systems according to manufacturer's protocols. The final cytokine concentration in supernatants of cultured cells was normalized to the amount of total DNA of the cultured cells.

### Transwell migration assays

The primary chondrocytes were transfected with a scramble miRNA (100 nM), *miR-33* mimic (100 nM) or anti-*miR-33* (100 nM) for 36 h. Then, the supernatant was collected as conditional medium (CM) for the chemotaxis test of monocytes in the transwell migration assays. The CCL2 antibody (anti-CCL2, 0.1 μg/ml) was used to block the effect of CCL2 in the CM. The human monocytes THP-1 were cultured and resuspended at 1×10^5^/ml. In migration assay, 200 μl of cell suspension was sucked into each insert of the transwell (PC membrane with 8.0 μm pore size; No. 3422, BD). The lower chambers were loaded with different kinds of CMs (4.2 ml). After culture for 2 h, the upper inserts were fixed with paraformaldehyde for 20 min, and then stained with 0.1% crystal violet. Five fields of view in the undersides of the membranes were randomly selected to count cells under a microscope (×200). The cell migration rate was described as the relative cell numbers of the transmitting cells.

### Statistics

All data were expressed as mean±S.E.M. and were analysed using either one-way ANOVA or two-tailed unpaired Student's *t* test. The difference between the groups was considered statistically significant for *P*<0.05. For each parameter of all data presented, *P<0.05 and **P<0.01.

## RESULTS

### Prediction of potential miRNAs targeting at the 3′UTR of *CCL2* gene

**Figure 1:**
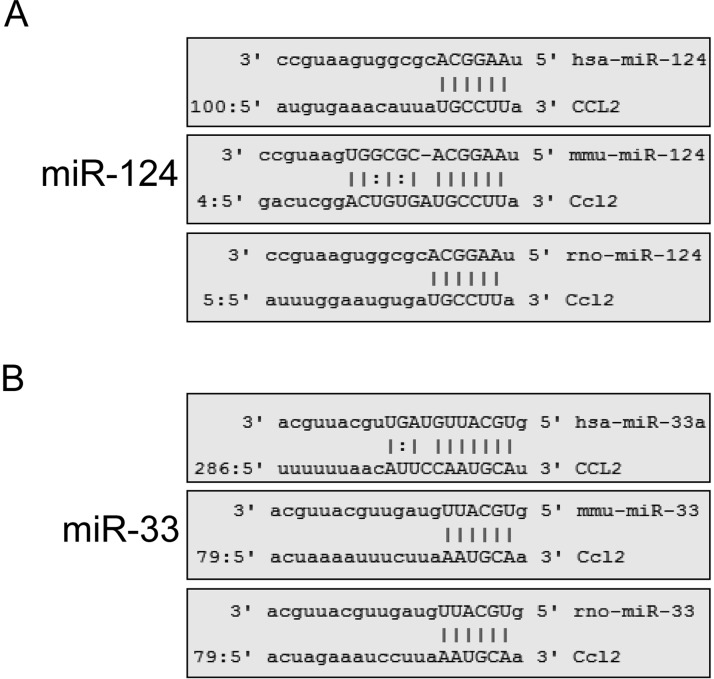
Prediction of potential miRNAs targeting at the 3′UTR of *CCL2* gene (**A** and **B**) The potential target sites of *miR-124* (A) or *miR-33* (B) in the 3′UTR of *CCL2* gene were conserved in human, mouse and rat species. The potential miRNAs targeting at the 3′UTR of *CCL2* genes in different species were predicted according to an online software. The conserved miRNAs were picked out.

To explore the miRNAs which could directly target at the 3′UTR of the *CCL2* gene, we predicted all the potential miRNAs in the 3′UTR of *CCL2* gene in human, mouse and rat species according to an online software (http://www.microrna.org/microrna/getGeneForm.do). We found that the potential binding sites for *miR-124* (Figure 1A) and *miR-33* (Figure 1B) were conserved in multiple species, indicating that those two miRNAs might be functional in CCL2 suppression. Indeed, *miR-124* was a well-documented suppressor of CCL2 in recent studies [20,21]. However, the role of *miR-33* in CCL2 expression was still not clear.

### *miR-33* is a regulator of CCL2 expression

**Figure 2:**
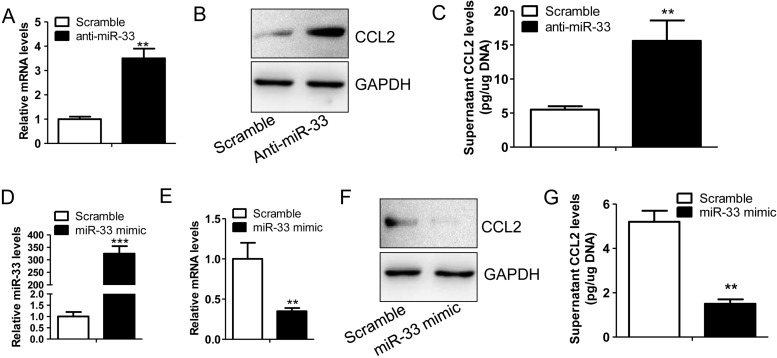
*miR-33* is a regulator of CCL2 expression (**A**) The relative mRNA levels of CCL2 in the primary mouse chondrocytes transfected with a scramble miRNA (100 nM) or an anti-miRNA of *miR-33* (100 nM) (*n*=4, **P<0.01). (**B**) Immunoblotting assay of CCL2 protein in the chondrocytes described in (A). (**C**) CCL2 levels in the supernatant of the chondrocytes described in (A) (*n*=4, **P<0.01). (**D**) The relative *miR-33* levels in the primary mouse chondrocytes transfected with a scramble miRNA (100 nM) or a *miR-33* mimic (100 nM) (*n*=4, ***P<0.005). (**E**) The relative mRNA levels of CCL2 in the primary mouse chondrocytes treated with a scramble miRNA (100 nM) or a *miR-33* mimic (100 nM) (*n*=4, **P<0.01). (**F**) Immunoblotting assay of CCL2 protein in the chondrocytes described in (E). (**G**) CCL2 levels in the supernatant of the chondrocytes described in (E) (*n*=4, **P<0.01).

To observe the regulatory role of *miR-33* in CCL2 expression, we performed gain- or loss-of-function studies on *miR-33* in primary chondrocytes. We demonstrated that the treatment with an anti-miRNA of *miR-33* could strikingly induce the expression of CCL2 in the mRNA (Figure 2A) and protein (Figure 2B) levels as well as the secretion of CCL2 in the supernatant of cultured chondrocytes (Figure 2C). Further, we overexpressed *miR-33* in chondrocytes by treating the cells with a *miR-33* mimic (Figure 2D). We found that treatment of *miR-33* mimic largely attenuated the mRNA (Figure 2E), protein (Figure 2F) and secretion (Figure 2G) levels of CCL2 in the chondrocytes. Those findings confirmed the suppressive role of *miR-33* in CCL2 expression.

### *miR-33* suppresses *CCL2* expression via targeting at the 3′UTR

**Figure 3:**
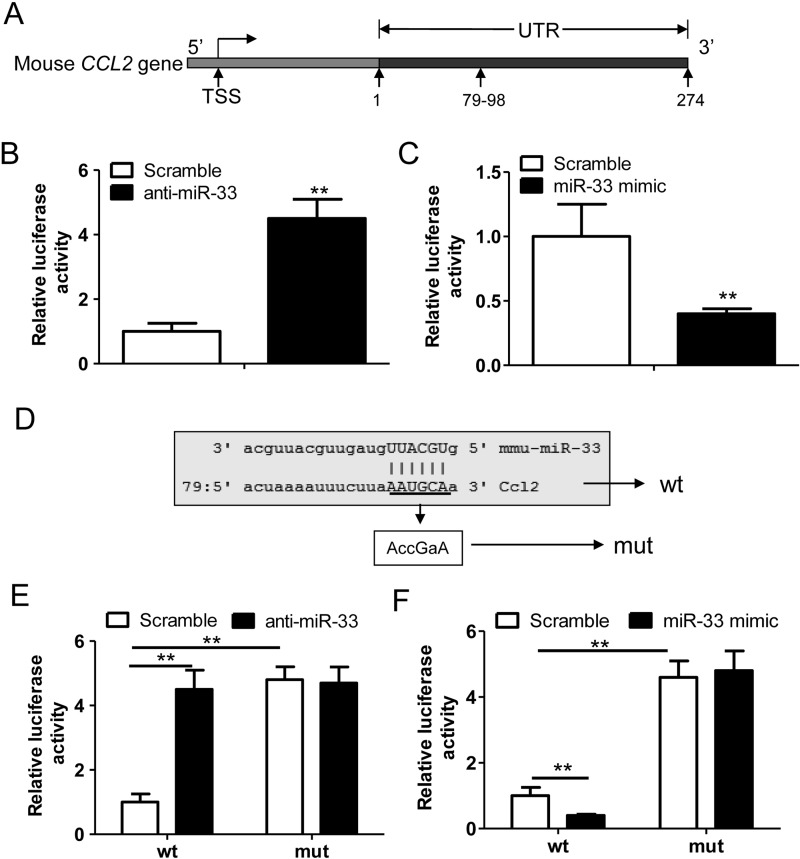
miR-33 suppresses *CCL2* expression via targeting at the 3′UTR (**A**) The schematic diagram of potential binding sites (79–98) for *miR-33* in the 3′UTR of mouse *CCL2* gene. (**B**) Relative luciferase activity of the chondrocytes transfected with a scramble miRNA (100 nM) or an anti-miRNA of *miR-33* (100 nM) plus the miRNA reporter plasmid (0.5 μg/ml) harbouring the 3′UTR of mouse *CCL2* gene (*n*=3, **P<0.01). (**C**) Relative luciferase activity of the chondrocytes transfected with a scramble miRNA (100 nM) or a mimic of *miR-33* (100 nM) plus the miRNA reporter plasmid (0.5 μg/ml) harbouring the 3′UTR of mouse *CCL2* gene (*n*=3, **P<0.01). (**D**) The potential binding sites AAUGCA (wt) of *miR-33* in the 3′UTR of *CCL2* gene was mutated as ACCGAA (mut). The 3′UTRs containing the wt or mut sites were subcloned into a miRNA reporter vector. (**E**) Relative luciferase activity of the chondrocytes transfected with a scramble miRNA (100 nM) or an anti-miRNA of *miR-33* (100 nM) plus the wt or mut miRNA reporter gene (*n*=3, **P<0.01). (**F**) Relative luciferase activity of the chondrocytes transfected with a scramble miRNA (100 nM) or *miR-33* mimic (100 nM) plus the wt or mut miRNA reporter gene (*n*=3, **P<0.01).

To investigate the precise regulatory mechanism between *miR-33* and CCL2, we subcloned the 3′UTR of mouse *CCL2* gene into a miRNA reporter gene vector (Figure 3A). As expected, we found that anti-*miR-33* treatment largely potentiated the reporter gene activity (Figure 3B), whereas *miR-33* mimic could significantly suppress the luciferase activity (Figure 3C). To further confirm the regulatory effect of *miR-33* on CCL2 via the 3′UTR, the potential binding sites AAUGCA were mutated as ACCGAA (Figure 3D). Noteworthily, the anti-*miR-33*-stimulated reporter gene activity was abolished with the mutation of the potential *miR-33* binding sites (Figure 3E). Identical results were obtained in response to the treatment of *miR-33* mimic (Figure 3F). Those results indicated that *miR-33* suppressed mouse CCL2 expression via a binding element locating at 79/98 in the 3′UTR.

### The *miR-33*/CCL2 axis in chondrocytes regulates the monocyte chemotaxis

**Figure 4:**
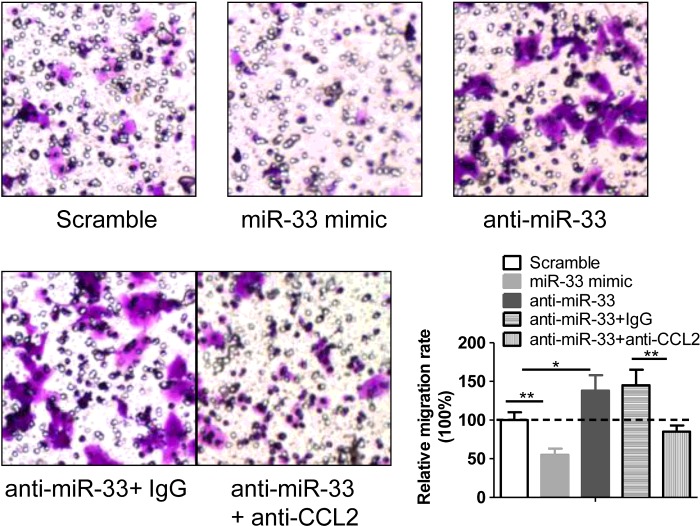
The *miR-33*/CCL2 axis in chondrocytes regulates the monocyte chemotaxis The primary chondrocytes were transfected with a scramble miRNA (100 nM), *miR-33* mimic (100 nM) or anti-*miR-33* (100 nM) for 36 h. Then, the supernatant was collected as CM for the chemotaxis test of monocytes in the transwell migration assays. The CCL2 antibody (anti-CCL2, 0.1 μg/ml) was used to block the effect of CCL2 in the medium. The representative images were displayed and the relative migration rate was calculated (*n*=6, *P<0.05 and **P<0.01).

CCL2 is a potent attractor of monocytes [12]. To observe the regulatory role of *miR-33*/CCL2 axis in monocyte chemotaxis, transwell migration assays were carried out. As shown in Figure 4, we found that the CM from *miR-33* mimic-treated chondrocytes significantly inhibited the migration rate of the monocytes, whereas the CM from anti-*miR-33*-treated chondrocytes notably stimulated the monocyte chemotaxis. Meanwhile, we demonstrated that the anti-*miR-33* treatment-induced monocyte chemotaxis was prevented by supplementary CCL2 antibody. Those findings indicated that *miR-33*/CCL2 axis in chondrocytes was functional in regulating monocyte chemotaxis.

### Decreased *miR-33* levels and elevated CCL2 levels in the cartilage of OA patients

**Figure 5:**
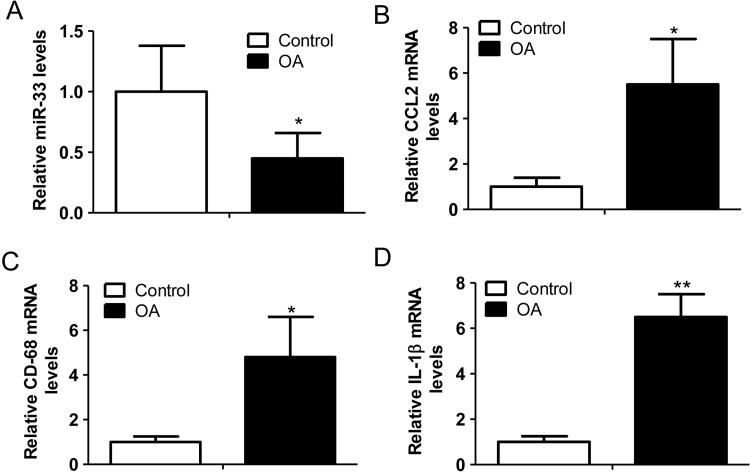
Decreased *miR-33* levels and elevated CCL2 levels in the cartilage of OA patients (**A**) Relative *miR-33* levels in the cartilages of OA patients compared with the ones of traumatic patients (control) (*n*=6–10, *P<0.05). (**B**) Relative CCL2 mRNA levels in the samples described in (A) (*n*=6–10, *P<0.05). (**C**) Relative CD-68 mRNA levels in the samples described in (A) (*n*=6–10, *P<0.05). (**D**) Relative IL-1β mRNA levels in the samples described in (A) (*n*=6–10, **P<0.01).

To further correlate our *in vitro* findings to the physiopathological condition, we determined the levels of *miR-33*, CCL2, CD-68 and IL-1β in the cartilage of the patients with OA. We demonstrated that the level of *miR-33* (Figure 5A) was decreased and the level of CCL2 (Figure 5B) was increased in the cartilage of OA patients compared with the control group. Meanwhile, we found that the level of CD-68, the surface marker of macrophages, was elevated in cartilage of OA subjects, indicating the increased recruitment of macrophages in OA condition (Figure 5C). Finally, we also verified the increased inflammation response in OA patients by showing the increased IL-1β mRNA levels in the cartilage (Figure 5D).

## DISCUSSION

In the present study, we are the first to report the expression of *miR-33* in mouse chondrocytes and identify CCL2 as its direct target. The *miR-33*/CCL2 axis plays an important role in regulating monocyte chemotaxis. These findings provided a potential mechanism of macrophages infiltration in articular tissues of OA patients.

The miRNAs are small non-coding RNAs that bind to complementary sequences in the 3′UTR of target mRNAs and contribute to gene regulation by reducing mRNA translation or destabilizing transcripts [24–26]. Recent work has shown that miRNAs have multiple effects in various tissues including the chondrocytes or the articular cartilage [17,27]. We recently reported that reciprocal inhibition between *miR-26a* and NF-κB downstream of saturated non-esterified fatty acid (NEFA) signal regulated obesity-related chronic inflammation in chondrocytes [17]. Here in chondrocytes, we identified *miR-33* as a regulator of CCL2 expression and monocyte chemotaxis. The previous and recent studies mainly focused the functions of *miR-33* on the cholesterol homoeostasis [28,29] and energy metabolism [30]. Therefore, our findings indicated a potential role of *miR-33* in OA. We presumed that the deficiency of *miR-33* in the chondrocytes of OA patients would potentiate the production of CCL2, which then attracted the monocytes from peripheric blood to the articular tissues. Those infiltrated macrophages would amplify the inflammation response in OA. However, the precise phenotype and mechanisms need to be proved *in vivo* in the future studies.

The *miR-33*/CCL2 axis was identified in the primary mouse chondrocytes in the present study. However, whether this axis exists ubiquitously in other cell types was still not elucidated. In a previous study, *miR-33* was reported to potentiate the pro-inflammatory activation of macrophages and aggravate the progression of atherosclerosis [31]. These findings implied that the *miR-33*/CCL2 axis might not exist in macrophages or that the function of *miR-33*/CCL2 axis might be antagonized by other *miR-33*-initiated factors. Therefore, further studies on the role of *miR-33*/CCL2 axis in other cell types might be very interesting.

In the present study, we predicted and selected out the potential miRNAs of CCL2 by choosing the conserved ones, *miR-124* and *miR-133*. Generally, the conserved miRNAs are most likely functional ones. It should be pointed out that the non-conservative miRNAs between the species can also be functional. The high-throughput screening technology should be used to select out those functional miRNAs besides *miR-124* and *miR-133* in the future.

Taken together, we identified *miR-133* as a novel suppressor of CCL2 in chondrocytes. The *miR-33*/CCL2 axis might regulate monocyte chemotaxis in OA. Blocking this axis might be a potential therapeutic strategy for the treatment of OA.

## Abbreviations

CM: conditional medium
MCP-1: monocyte chemoattractant protein-1
OA: osteoarthritis
